# Efficacy of Ketogenic Diet for Infantile Spasms in Chinese Patients With or Without Monogenic Etiology

**DOI:** 10.3389/fped.2022.842666

**Published:** 2022-03-17

**Authors:** Jun Wang, Jie Zhang, Ying Yang, Kai Gao, Ye Wu, Yuehua Zhang, Yuwu Jiang

**Affiliations:** ^1^Department of Pediatrics, Peking University First Hospital, Beijing, China; ^2^Department of Neurology, Children's Hospital Affiliated to Capital Institute of Pediatrics, Beijing, China; ^3^Beijing Key Laboratory of Molecular Diagnosis and Study on Pediatric Genetic Diseases, Beijing, China; ^4^Children Epilepsy Center, Peking University First Hospital, Beijing, China; ^5^Center of Epilepsy, Beijing Institute for Brain Disorders, Beijing, China

**Keywords:** ketogenic diet, infantile spasms, whole-exome sequencing, *CDKL5* gene, monogenic etiology

## Abstract

**Objective:**

The aim of this study was to evaluate the efficacy of the ketogenic diet (KD) for infantile spasms (IS) in patients with and without different causative genetic mutations.

**Methods:**

We retrospectively evaluated the data of 119 infants with IS who underwent whole-exome sequencing (WES) before KD treatment. The KD efficacy was analyzed at the 16th week after initiation. Patients showing ≥ 50% seizure reduction from baseline and/or the disappeared hypsarrhythmia were considered as the responders. Chi-squared tests or two-sided Fisher's exact tests were performed for categorical data and Mann–Whitney *U*-tests for non-parametric and continuous data.

**Results:**

The responder rate to KD in 119 patients was 47.90%. Six different causative monogenic mutations were identified in 32 (26.89%) patients with IS, including *CDKL5* (*n* = 8), *ALG13* (*n* = 3), *KCNT1* (*n* = 8), *SLC35A2* (*n* = 5), *PCDH19* (*n* = 4), and *STXBP1* (*n* = 4). Patients with *CDKL5* mutations showed a significantly better response to KD (87.50%) than patients without *CDKL5* mutations (*p* = 0.03). Seven of eight patients with *CDKL5* mutations were responders, including five mutations located in functional motifs, and two mutations in the catalytic domain.

**Conclusion:**

KD therapy was effective in infants with IS. Patients with *CDKL5* mutations might have a better response to KD treatment.

## Introduction

Infantile spasms (IS) is a specific type of seizure seen in an epilepsy syndrome of infancy and childhood, which is characterized by epileptic spasms, developmental problems, and a specific brain wave pattern on electroencephalography (EEG) testing called hypsarrhythmia (1). To date, the treatment of infantile spasms is challenging. First-line treatment options-namely hormonal therapy and vigabatrin-display moderate to high efficacy but also exhibit substantial side-effect burdens. Although surgical resection is a well-established treatment option for highly selected patients with IS, the ideal sequence of treatment is unknown. Among non-pharmacologic therapies, the greatest attention has been focused on the ketogenic diet. Many retrospective studies have shown that the ketogenic diet is effective in the treatment of IS, suggesting that the ketogenic diet has the potential to be used as alternated treatment to hormone therapy or VGB (2). Therefore, KD, as a high-fat, adequate-protein, and low-carbohydrate diet, has become a safer and more effective alternative treatment option for intractable childhood epilepsy than drugs and surgery since 1921 (3), based on the hypothesis that the simulation of metabolic starvation can induce anti-seizure effects *via* the production of ketone bodies (4). However, the restrictive diet regimen and potential side effects of KD limit the medical application of KD (5).

Diverse genetic mutations, especially *de novo* monogenic mutations, are found to constitute a significant portion of the etiologies of IS, owing to advances in genetic testing technologies (e.g., whole-exome sequencing) (6). The discovery of pathogenic genes for IS has improved our understanding of the pathophysiology of the disease. However, the knowledge about the correlation between genotype in patients with infantile spasm and effective KD therapy was quite limited. Some studies have shown that KD can be effective in treating certain epilepsy syndromes such as infantile spasms and Dravet syndrome. However, one epilepsy syndrome can be caused by different genetic variants, due to genetic heterogeneity (7). It would be difficult to predicting if patients were responsive to KD therapy. Therefore, this study aimed to assess the efficacy of KD therapy for IS patients caused by different monogenic genes.

## Materials and Methods

### Patients

Here, we evaluated the efficacy of a 16-week ketogenic diet (KD) in IS patients with different pathogenic gene mutations. Totally 119 IS patients by KD treatment with prior Whole-exome sequencing (WES) test results were collected in this study in the Pediatric Department of Peking University First Hospital in China, from November 2017 to December 2018. This study was approved by the Institutional Review Board of Peking University First Hospital. Parental written informed consent was obtained for all patients included in this study.

Inclusion criteria were as follows: (1) pediatric patients diagnosed with IS exhibiting characteristic EEG pattern of hypsarrhythmia and delayed social, behavioral, and other cognitive development; (2) patients who failed to achieve seizure freedom or prevent recurrence after adrenocorticotrophic hormone/glucocorticoid and who stopped hormone therapy for least 14 days before KD initiation; (3) patients who failed to achieve seizure freedom with adequate trials of two or more anti-seizure medications; (4) patients whose seizures and developmental delays were noticed before the age of 3 years patients who underwent WES; (5) patients continued the KD for at least 16-weeks. Exclusion criteria were as follows: (1) patients with proven etiologies other than genetic etiology (e.g., secondary structural deformities, and infectious, or immune encephalopathies); (2) patient with possibly resectable lesion by surgery.

### WES and Bioinformatic Analyses

Genomic DNA was extracted from peripheral blood samples by using a blood and tissue genomic DNA isolation kit (TianGen DP349), according to the manufacturer's instructions. For WES analysis, genomic DNA fragments were captured by using the SSEL XT Human All Exon v6 kit (Agilent Technologies, Santa Clara, CA, U.S.A.) and sequenced on the NovaSeq 6000 (Illumina, San Diego, CA, U.S.A.) sequencing platform with 150 bp paired-end reads. Raw data were transformed to fastq file by bcl2fastq inhouse package (Illumina). Exome data processing, sequence alignment to GRCh38/hg38 reference genome, variant calling, and variant annotation were performed by using BWA, SAMtools and Pica. Annotations of genetic variants were performed by ANNOVAR. Further interpretation regarding pathogenic, likely pathogenic, or variant of uncertain significance was performed following the criteria of American College of Medical Genetics (ACMG), and further filtration was performed following two rules: (1) target genes were associated with the patients' phenotypes; (2) target genes were congregation in Trios.

### Ketogenic Diet Intervention

Patients were instructed to follow a classic 3:1 KD diet by the attending pediatric epileptologists (8). According to the patient's age, height, and body weight, the recommended daily intake was calculated. Calories were restricted to 75% of the recommended daily intake. The patients were supplemented with daily required amounts of calcium, vitamins, and minerals, without sucrose and lactose. During KD treatment, the dosage of the primary anti-seizure medications was kept unchanged.

### Clinical Observation

Complete clinical data included detailed documentation of seizure frequencies and EEGs (at least for 4 h including awake and sleep phase). For the assessment of efficacy data, EEGs was recorded before and 16-weeks after the KD treatment. EEGs were scored according to the burden of amplitudes and epileptiform discharges (9).

The reduction in spasms and remission of hypsarrhythmia in EEG after the 16-weeks of treatment was used to indicate KD efficacy. The result was grouped into grade I (electro-clinical remission): epileptic spasms were completely controlled for at least 1-week with remission of hypsarrhythmia in EEG; grade II (partially effective): the frequency of epileptic spasms reduced by more than 50% and/or the remission of hypsarrhythmia in EEG; and grade III (ineffective): the frequency of epileptic spasms reduced by <50% with hypsarrhythmia. Patients after 16-week KD had definite efficacy data were classified as responder (grade I+II) or non-responder (grade III).

### Statistical Analysis

Comparisons between two groups were performed using chi-square tests or two-sided Fisher's exact tests for categorical data and using Mann–Whitney *U*-tests for non-parametric and continuous data. All statistical analyses were done with SPSS 26.0 (SPSS Inc., Chicago, IL, USA). Data were expressed as the median with the interquartile range (IQR) or as the number and percentage, and the data with *p* < 0.05 were considered statistically significant.

## Results

### Patients and Clinical Characteristics

Patients were divided into responders (grade I+II) and non-responders (grade III) groups (Table 1). Clinical variables included age at seizure onset, age at KD initiation, lead time from seizure onset to KD initiation, gender, and identified genetic etiology. The median age of patients at seizure onset and KD initiation were 5.00 months (IQR = 3.00–8.00 months) and 17.00 months (IQR = 11.00–23.00 months), respectively. And the medium lead time from seizure onset to KD initiation was 10.00 months (IQR = 6.00–16.00 months). Of the 119 patients, 57 (47.90%) patients were considered as the KD responders, while the other 62 (52.10%) patients didn't have obvious benefit from the 16-week KD treatment. No statistical differences were found between responders and non-responders with respect to clinical variables including the age at seizure onset, age at KD initiation, lead time from seizure onset to KD and genetic mutations. However, there was a significant difference between KD responders and non-responders in terms of gender (*p* = 0.04), i.e., females tended to respond better to the KD treatment. In addition, there was no significant association between whether a patient carried a IS-related mutation and their response to KD treatment (*p* = 0.89).

**Table 1:** Demographics of patients and comparison between responders and non-responders after 16-week KD treatment (3:1).

	**Total** **(*n* = 119)**	Responders^*^ **(*n* = 57, 47.90%)**	**Non-responders** ***n* = 62, 52.10%)**	***p***
Age at seizure onset (months)	5.00 (3.00–8.00)	5.00 (3.00–8.00)	5.00 (3.00–7.25)	0.75^a^
Age at KD treatment (months)	17.00 (11.00–23.00)	15.00 (11.00–22.00)	18.50 (11.00–24.25)	0.07^a^
Lead time from seizure onset to KD (months)	10.00 (6.00–16.00)	9.00 (5.75–15.00)	12.00 (6.00–17.00)	0.06^a^
**Gender**				0.04^b^
Male	76	31 (40.79%)	45 (59.21%)	
Female	43	26 (60.47%)	17 (39.53%)	
**Genetic etiology**				0.89^b^
Unknown	87	42 (48.28%)	45 (51.72%)	
Known	32	15 (46.88%)	17 (53.12%)	

*Data are presented as the median (interquartile range) or as the number (percent)*.

*KD, ketogenic diet*.

* *Responders to KD represent patients who showed ≥ 50% seizure reduction from baseline*.

a *Mann–Whitney U-tests*.

b *chi-squared tests*.

We also analyzed clinical variables between patients with and without IS-related mutations. The etiology of patients without IS-related mutations in this study was considered idiopathic. As shown in Table 2, there were no significant differences in terms of age at KD initiation and lead time from seizure onset to KD. However, among patients with identified mutations, the age at seizure onset was younger (3.25 months, *p* = 0.01) and the proportion of females was higher (41.86%, *p* = 0.01) compared to patients without pathogenic mutations.

**Table 2:** Demographics of patients and comparison between patient with and without identified mutations at 16-weeks after KD (3:1) initiation.

	**Total** **(*n* = 119)**	**Identified mutations** **(*n* = 32, 26.89%)**	**Without identified mutations** **(*n* = 87, 73.11%)**	***p***
Age at seizure onset, months	5.00 (3.00–8.00)	3.25 (1.35–6.00)	6.00 (4.00–8.00)	0.01^a^
Age at KD initiation	17.00 (11.00–23.00)	12.50 (7.00–24.25)	18 (12.00–23.00)	0.08^a^
Lead time from seizure onset to KD (months)	10.00 (6.00–16.00)	5.43 (9.00–17.00)	11.00 (6.00–16.00)	0.58^a^
**Gender**				0.01^b^
Male	76	14 (18.42%)	62 (81.58%)	
Female	43	18 (41.86%)	25 (58.14%)	

*Data are presented as the median (interquartile range) or as the number (percent)*.

*KD, ketogenic diet*.

a *Mann–Whitney U-tests*.

b *chi-squared tests*.

### Causative Monogenic Mutations

**Figure 1:**
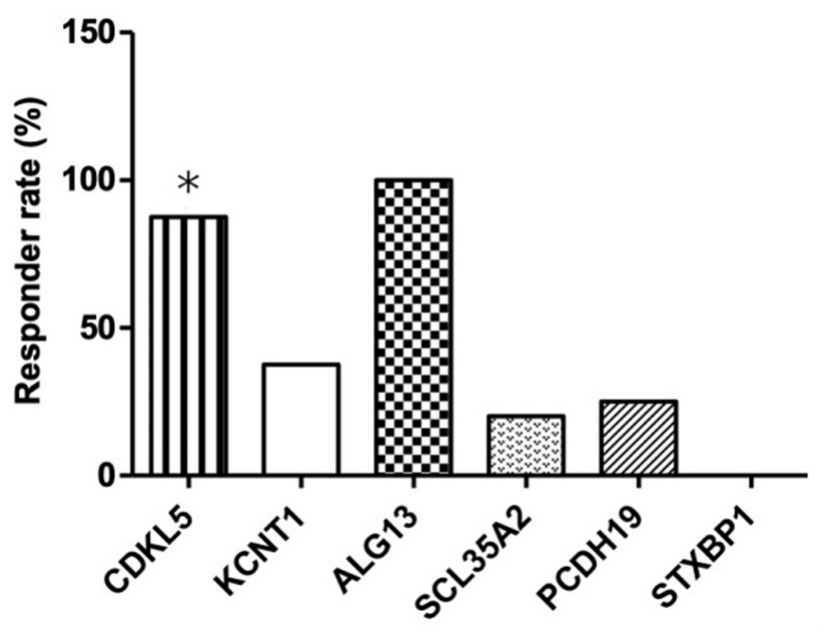
Responder rates to 16-week KD treatment according to the identified pathogenic genetic mutations in patients with IS. *Responder rates that are significantly higher than the non-responder rate in patients with *CDKL5* mutation.

As shown in Table 3, six different causative monogenic mutations were identified in 32 (26.89%) patients with IS. Amongst them, 15 (46.88%) patients were responders and the other 17 (53.12%) were considered as non-responders to KD therapy. The most common mutations were in the cyclin dependent kinase like 5 (*CDKL5*) gene (*n* = 8 [25.00%]) and potassium sodium-activated channel subfamily T member 1 (*KCNT1*) gene (*n* = 8 [25.00%]), followed by solute carrier family 35 member A2 (*SLC35A2*) (*n* = 5 [15.63%]), protocadherin 19 (*PCDH19*) (*n* = 4 [12.50%]), syntaxin binding protein 1 (*STXBP1*) (*n* = 4 [12.50%]) and asparagine-linked glycosylation 13 (*ALG13*) gene (*n* = 3 [9.37%]). To explore the relation between efficacy of KD and causative monogenic gene, the patients were classified as responder (grade I+II) or non-responder (grade III) group. In order to evaluate the efficacy of KD in infantile spasms (IS) with one specific mutated gene, the efficacy of KD in patients with each specific mutated gene was compared with that of those patients without the mutation in that gene. Responder rate to KD of the patients with mutations in *CDKL5* (responder rate = 87.50%; *p* = 0.03) was significantly higher than responder rate of the patients without *CDKL5* mutation. Responder rate of patients with *ALG13*mutations (responder rate = 100%; *p* = 0.11) was higher than responder rate of patients without *ALG3* mutation, and responder rate of patients with mutations in *KCNT1* (responder rate = 37.5%; *p* = 0.72), *SLC35A2* (responder rate = 20.00%; *p* = 0.37), *PCDH19* (responder rate = 25.00%; *p* = 0.62) or *STXBP1* (responder rate = 0; *p* = 0.12) was lower than responder rate of patients without the respective gene mutations (Table 3 and Figure 1).

**Table 3:** Responses to 16-week KD (3:1) treatment related to specific mutated gene, with the *p*-value for the comparison of the efficacy of KD in patients with each specific mutated gene to that of those patients without the mutation in that specific gene.

**Pathogenic gene** **(*n* = 32)**	Responders^*^ **(*n* = 57, 47.90%)**	**Non-responders** **(*n* = 62, 52.10%)**	***p***
*CDKL5* (*n* = 8)	7 (87.50%)	1 (12.50%)	0.03^a^
*KCNT1* (*n* = 8)	3 (37.50%)	5 (62.5%)	0.72^a^
*ALG13* (*n* = 3)	3 (100.00%)	0	0.11^a^
*SLC35A2* (*n* = 5)	1 (20.00%)	4 (80.00%)	0.37^a^
*PCDH19* (*n* = 4)	1 (25.00%)	3 (75.00%)	0.62^a^
*STXBP1* (*n* = 4)	0	4 (100%)	0.12^a^

* *Responders to KD represent patients who showed ≥ 50% seizure reduction from baseline*.

a *Two-sided Fisher's exact tests*.

**Figure 2:**
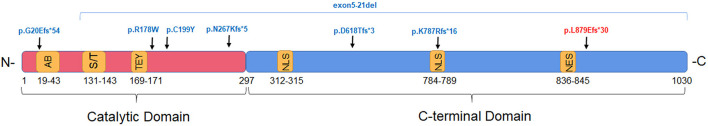
The schematic graph indicating the locations of *CDKL5* variants in functional domains in patient with IS. *CDKL5* mutations identified in responders and non- responders to KD treatment are indicated in blue and red, respectively. Functional motifs are labeled in yellow: AB, ATP binding site; S/T, Ser/Thr kinase active site; TEY, Thr/Glu/Tyr motif; NLS, nuclear localization signal; NES, nuclear export signal.

In our cohort, 31 patients presented with *de novo* mutations, and one mutation in the *ALG13* gene was inherited from the respective patient's mother. The *CDKL5* mutations located in the exon 2, 8, 9, 10, 12, 16, or 18. In addition, among eight patients with *CDKL5* genetic mutations, seven patients (87.50%) responded to KD therapy. Furthermore, among the *CDKL5* coding mutations, five of them were truncation mutations that directly influenced different functional motifs of CDKL5 protein, such as the ATP binding site, Ser/Thr kinase active site, Thr/Glu/Tyr motif, nuclear localization signal (NLS), and nuclear export signal (NES). And the other 2 mutations were missense mutations in the catalytic domain. One patient whose mutation was truncation without loss of the functional motifs did not respond to KD treatment (Figure 2). Moreover, all genetic variations in *ALG13* in three patients were clustered in exon 2 or 3, and all three patients were responders to KD treatment. But all four patients with *STXBP1* variations did not respond to KD treatment. Genetic features of other causative monogenic mutations are shown in Table 4. Taken together, these results indicated that different IS-associated genetic mutations had a differential influence on the patient's response to KD therapy, suggesting the involvement of diverse disease mechanisms in the onset of IS.

**Table 4:** Genotypic features of patients with identified IS-related gene variants.

**Case**	**Variant (NM_003159)**	**Evidence for Pity based on ACMG guideline**				**Category**	**Responder (+)**
	***CDKL5***	**Very strong**	**Strong**	**Moderate**	**Supporting**		**Non-responder (–)**
1/F	c.1851delT (p.D618Tfs*3)	PVS1	PS2	PM2		P	+
2/M	c.58_64delAGGTGAAGinsA (p.G20fs*54)	PVS1	PS2	PM2		P	+
3/F	c.2356delA (p.K787Rfs*16)	PVS1	PS2	PM2		P	–
4/F	exon5–21del	PVS1	PS2	PM2		P	+
5/F	c.2635_2636del (p.L879fs*30)	PVS1	PS2	PM2		P	+
6/F	c.596G>A (p.C199Y)		PS2	PM1+PM2	PP3	LP	+
7/F	c.532C>T (p.R178W)		PS2	PM1+PM2	PP3	LP	+
8/M	c.800_801del (p.N267fs*5)	PVS1	PS2	PM2		P	+
**Case**	**Variant (NM_001099922)**	**Evidence for Pity based on ACMG guideline**				**Category**	**Responder (+)**
	***ALG13***	**Very strong**	**Strong**	**Moderate**	**Supporting**		**Non-responder (–)**
1/F	c.320A>G (p.N107S)		PS2	PM2	PP3	LP	+
2/F							+
3/F	c.241G>A (p.A81T)		PS2	PM2	PP3	LP	+
**Case**	**Variant (NM_003165)**	**Evidence for Pity based on ACMG guideline**				**Category**	**Responder (+)**
	***STXBP1***	**Very strong**	**Strong**	**Moderate**	**Supporting**		**Non-responder (–)**
1/M	c.548T>C (p.L183P)		PS2	PM1+PM2	PP2+PP3	LP	–
2/M	c.164T>C (p.I55T)		PS2	PM1+PM2	PP2+PP3	LP	–
3/M	c.1439C>T (p.P480L)		PS2	PM1+PM2	PP2+PP3	LP	–
4/F	c.124_126delTCC (p.42delS)		PS2	PM2+PM4		LP	–
**Case**	**Variant (NM_005660)**	**Evidence for Pity based on ACMG guideline**				**Category**	**Responder (+)**
	***SLC35A2***	**Very strong**	**Strong**	**Moderate**	**Supporting**		**Non-responder (–)**
1/M	c.221C>T (p.A74V)		PS2	PM2	PP3	LP	–
2/F	c.78delG (p.T27fs*29)	PVS1	PS2	PM2		P	–
3/F	c.426+1G>A	PVS1	PS2	PM2		P	–
4/F	c.128T>C (p.L43P)		PS2	PM2	PP3	LP	+
5/F	c.692dupT (p.W232Vfs*23)		PVS1-Strong+PS2	PM2		P	–
**Case**	**Variant (NM_020766)**	**Evidence for Pity based on ACMG guideline**				**Category**	**Responder (+)**
	***PCDH19***	**Very strong**	**Strong**	**Moderate**	**Supporting**		**Non-responder (–)**
1/F	c.1225G>T (p.G409*)	PVS1	PS2	PM2		P	–
2/F	c.1544_1577del (p.I515fs*44)	PVS1	PS2	PM2		P	+
3/M	c.317T>A (p.M106K)		PS2	PM2	PP3	LP	–
4/M	c.1639G>C (p.A547P)		PS2	PM1+PM2	PP3	LP	–
**Case**	**Variant (NM_020822)**	**Evidence for Pity based on ACMG guideline**				**Category**	**Responder (+)**
	***KCNT1***	**Very strong**	**Strong**	**Moderate**	**Supporting**		**Non-responder (–)**
1/M	c.862G>A (p.G288S)		PS2	PM1+PM2	PP3	LP	–
2/M							+
3/M	c.1193G>A (p.R398Q)		PS2	PM2	PP3	LP	–
4/M							–
5/F	c.1421G>A (p.R474H)		PS2	PM1+PM2	PP3	LP	–
6/M	c.2800G>A (p.A934T)		PS2	PM1+PM2	PP3	LP	+
7/M							–
8/F	c.1225C>T (p.P409S)		PS2	PM2	PP3	LP	+

*P, Pathogenic; LP, likely pathogenic*.

## Discussion

KD has become increasingly popular as one of the non-pharmacological treatment options for pediatric patients with IS (10). In this study, 57 (47.90%) patients were considered as the KD responders, consistent with earlier KD treatment reports showing about 50% response for IS at 12 or 13 months (11, 12), which suggested that KD therapy could cure a subset of patients with IS. Here, the significant differences we observed between KD responders and non-responders in terms of gender are consistent with previous KD treatment for epilepsy studies (13). Female mice with epilepsy benefited more in KD, suggesting that differences in KD-treated mice may be related to gender-related β-hydroxybutyrate levels (14, 15). Thus, KD therapy might be more effective in girls than in boys. Although the median lead time of the responders' group and the non-responders' group was 9 and 12 months, respectively. The lead time from seizure onset to KD showed no significant difference with response to KD (*P* = 0.06), which might be related to the small sample size in this study.

WES has been widely utilized to detect the causative mutations in patients with developmental and epileptic encephalopathy, mental disorders, and IS (16). In this study, the molecular diagnostic rate was 26.89% for 119 patients with IS, which was consistent with previous studies (17, 18). In addition, the age at seizure onset and gender were significantly correlated with identified genes in this study. Thus, we deduced that disease-causing genetic mutations were more likely to occur in patients with early-onset IS. Moreover, the proportion of boys was significantly lower than girls in patients with identified mutations, suggesting that pathogenic gene mutations were more likely to occur in female patients with IS.

Understanding of the genetic etiology can be helpful to predict the efficacy of KD treatment for patients with IS (19). Prediction on patients who respond to KD therapy can be difficult. KD is a well-established treatment for patients with specific metabolic disorders, i.e., GLUT1 deficiency. In our study, there was no significant difference between the patient groups (with vs. without molecular diagnosis) in terms of response rate. However, the negative molecular diagnosis might be due to the technical limitations on the detection of the mutations in this study. KD therapy was considered to be the most effective way to treat patients with *SLC2A1* gene mutations. Unfortunately, we did not detect any such variant in our IS cohort. Therefore, the efficacy of KD in patients with each gene was compared with those of patients without the same gene. Patients with *CDKL5* mutations exhibited significantly favorable responses to KD treatment. Patients with *KCNT1, ALG13, SLC35A2, PCDH19*, or *STXBP1* mutations showed non-significantly favorable responses to KD compared to the responses without the same genetic mutations. This is consistent with the results from previous trials that showed a better response to KD in children with specific syndromes, especially those with *CDKL5* mutations (20, 21). Thus, the efficacy of KD was different in patients with various types of pathogenic mutations and was more effective in patients with *CDKL5* mutation-induced IS. CDKL5, as a serine-threonine kinase plays an important role in alternative splicing, neuronal morphogenesis, dendritic arborization, and energy metabolism (21). In this study, all mutations except for p.N267fs^*^5 produced proteins with presumably affected functional motifs, seven out of eight patients with these mutations responded effectively to KD treatment. Lim et al. (20) have found that patients harboring missense and/or in-frame variants within the catalytic domain tended to need a longer period of time for KD treatment to be effective than those carrying non-functional protein-truncating variants. These findings indicated that the KD was effective in patients with *CDKL5* mutations that could affect important motifs of CDKL5 protein (20, 22). Thus, the KD might be an optimal treatment for IS infants with *CDKL5* mutations.

All 3 cases with *ALG13* mutations responded effectively to KD. *ALG13* gene encodes a crucial protein involved in the process of N-linked glycosylation, and abnormal N-linked glycosylation leads to neurological deficits and disorders (23). In this study, although patients with *ALG13* mutations had non-significantly favorable responses to KD than those without *ALG13*, all three patients with *ALG13* mutations were responders to KD. Bobby et al. (24) reported that 9 out of 12 patients with *ALG13* mutations responded to KD treatment. KD treatment for IS patients with *ALG13* mutations was considered as an efficient therapy.

STXBP1 is an essential protein for presynaptic vesicle release. Four patients with *STXBP1* mutations in our study had no response to KD treatment. Li et al. (25) has reported a case with *STXBP1* mutations also had no response to a two-year KD treatment. Li et al. (26) reported three patients, of whom two failed to respond, and one female patient was seizure-free after KD treatment for 1-week. Therefore, it is suggested that KD therapy is not suitable for patients with *STXBP1* mutations.

Several limitations of this study should be noted, including the small sample size thus a few IS-related genes/variants were studied and short follow-up period. Therefore, additional prospective studies including a larger number and greater diversity of genes/variants, more complete clinical data, and long-term evaluation are required to confirm our findings.

## Conclusions

Collectively, our findings suggested that KD is an effective treatment for the patients with IS. Female patients exhibited a comparatively better response to KD therapy than male subjects. WES yielded a higher diagnostic rate among younger or female patients with IS. The KD treatment was particularly effective in patients with IS caused by *CDKL5* mutations influencing the protein kinase motifs. However, KD was not effective in patients with IS caused by *KCNT1, SLC35A2, PCDH19*, and *STXBP1* mutations. These results will provide rational evidence for effective treatment for patients with IS.

## Data Availability Statement

The datasets presented in this article are not readily available due to legal restrictions. Requests to access the datasets should be directed to the corresponding author.

## Ethics Statement

The studies involving human participants were reviewed and approved by Institutional Review Board of Peking University First Hospital. Written informed consent to participate in this study was provided by the participants' legal guardian/next of kin.

## Author Contributions

JW and YJ conceived and designed the experiment, reviewed, and edited the manuscript. JW carried out the initial analyses and drafted the initial manuscript. JZ, YY, YW, and YZ collected and analyzed data. KG drew the protein structure diagram. All authors approved the final version of the manuscript.

## Funding

This work was supported by the National Key Research and Development Program of China (grant numbers: 2020YFA0804000, 2020YFA0804003, and 2016YFC1306201), by the National Natural Science Foundation of China (grant numbers: 81971211, 12026606, and 81601131), by Beijing Natural Science Foundation (7212109), by the Beijing Key Laboratory of Molecular Diagnosis and Study on Pediatric Genetic Diseases (grant number: BZ0317), and by the Fundamental Research Funds for the Central Universities (grant numbers: BMU2017JI002, BMU2018XY006, and PKU2017LCX06).

## Conflict of Interest

The authors declare that the research was conducted in the absence of any commercial or financial relationships that could be construed as a potential conflictof interest.

## Publisher's Note

All claims expressed in this article are solely those of the authors and do not necessarily represent those of their affiliated organizations, or those of the publisher, the editors and the reviewers. Any product that may be evaluated in this article, or claim that may be made by its manufacturer, is not guaranteed or endorsed by the publisher.

## References

[1] Wong M Trevathan E. Infantile spasms. Pediatr Neurol. (2001) 24:89–98. 10.1016/S0887-8994(00)00238-1 11275456

[2] Hussain SA. Treatment of infantile spasms. Epilepsia Open. (2018) 3:143–54. 10.1002/epi4.12264 30564773 PMC6293071

[3] Sampaio LP. Ketogenic diet for epilepsy treatment. Arq Neuropsiquiatr. (2016) 74:842–8. 10.1590/0004-282X20160116 27759811

[4] Freeman JM Kossoff EH Hartman AL. The ketogenic diet: one decade later. Pediatrics. (2007) 119:535–43. 10.1542/peds.2006-2447 17332207

[5] Kang HC Chung DE Kim DW Kim HD. Early- and late-onset complications of the ketogenic diet for intractable epilepsy. Epilepsia. (2004) 45:1116–23. 10.1111/j.0013-9580.2004.10004.x 15329077

[6] Moller RS Dahl HA Helbig I. The contribution of next generation sequencing to epilepsy genetics. Expert Rev Mol Diagn. (2015) 15:1531–8. 10.1586/14737159.2015.1113132 26565596

[7] Eric HK Beth AZ Stephane A Karen RB Christina B Robyn B. Optimal clinical management of children receiving dietary therapies for epilepsy: updated recommendations of the International Ketogenic Diet Study Group. Epilepsia Open. (2018) 3:175–92. 10.1002/epi4.12225 29881797 PMC5983110

[8] Kossoff EH McGrogan JR Bluml RM Pillas DJ Rubenstein JE Vining EP. A modified Atkins diet is effective for the treatment of intractable pediatric epilepsy. Epilepsia. (2006) 47:421–4. 10.1111/j.1528-1167.2006.00438.x 16499770

[9] Mytinger JR Hussain SA Islam MP Millichap JJ Patel AD Ryan NR. Improving the inter-rater agreement of hypsarrhythmia using a simplified EEG grading scale for children with infantile spasms. Epilepsy Res. (2015) 116:93–8. 10.1016/j.eplepsyres.2015.07.008 26280806

[10] Velisek L Veliskova J. Modeling epileptic spasms during infancy: are we heading for the treatment yet? Pharmacol Ther. (2020) 212:107578. 10.1016/j.pharmthera.2020.107578 32417271 PMC7299814

[11] Kossoff EH Pyzik PL McGrogan JR Vining EP Freeman JM. Efficacy of the ketogenic diet for infantile spasms. Pediatrics. (2002) 109:780–3. 10.1542/peds.109.5.780 11986436

[12] Numis AL Yellen MB Chu-Shore CJ Pfeifer HH Thiele EA. The relationship of ketosis and growth to the efficacy of the ketogenic diet in infantile spasms. Epilepsy Res. (2011) 96:172–5. 10.1016/j.eplepsyres.2011.05.012 21700429

[13] Agarwal N Arkilo D Farooq O Gillogly C Kavak KS Weinstock A. Ketogenic diet: predictors of seizure control. SAGE Open Med. (2017) 5:2050312117712887. 10.1177/2050312117712887 28620490 PMC5464518

[14] Chun KC Ma SC Oh H Rho JM Kim DY. Ketogenic diet-induced extension of longevity in epileptic Kcna1-null mice is influenced by gender and age at treatment onset. Epilepsy Res. (2018) 140:53–55. 10.1016/j.eplepsyres.2017.11.005 29245026 PMC5826793

[15] Ren Y Chang J Li C Jia C Li P Wang Y. The effects of ketogenic diet treatment in kcna1-null mouse, a model of sudden unexpected death in epilepsy. Front Neurol. (2019) 10:744. 10.3389/fneur.2019.00744 31354612 PMC6635472

[16] Nabbout R Copioli C Chipaux M Chemaly N Desguerre I Dulac O. Ketogenic diet also benefits Dravet syndrome patients receiving stiripentol: a prospective pilot study. Epilepsia. (2011) 52:e54–57. 10.1111/j.1528-1167.2011.03107.x 21569025

[17] Mercimek-Mahmutoglu S Patel J Cordeiro D Hewson S Callen D Donner EJ. 3rd Diagnostic yield of genetic testing in epileptic encephalopathy in childhood. Epilepsia. (2015) 56:707–16. 10.1111/epi.12954 25818041

[18] Ko A Youn SE Kim SH Lee JS Kim S Choi JR. Targeted gene panel and genotype-phenotype correlation in children with developmental and epileptic encephalopathy. Epilepsy Res. (2018) 141:48–55. 10.1016/j.eplepsyres.2018.02.003 29455050

[19] Schoeler NE Cross JH Sander JW Sisodiya SM. Can we predict a favourable response to Ketogenic Diet Therapies for drug-resistant epilepsy? Epilepsy Res. (2013) 106:1–6. 10.1016/j.eplepsyres.2013.06.002 23820448

[20] Lim Z Wong K Olson HE Bergin AM Downs J Leonard H. Use of the ketogenic diet to manage refractory epilepsy in CDKL5 disorder: experience of >100 patients. Epilepsia. (2017) 58:1415–22. 10.1111/epi.13813 28605011

[21] Amin S Majumdar A Mallick AA Patel J Scatchard R Partridge CA. Caregiver's perception of epilepsy treatment, quality of life and comorbidities in an international cohort of CDKL5 patients. Hippokratia. (2017) 21:130–5. 10.1016/j.ejpn.2017.04.1141 30479474 PMC6247997

[22] Moseley BD Dhamija R Wirrell EC Nickels KC. Historic, clinical, and prognostic features of epileptic encephalopathies caused by CDKL5 mutations. Pediatr Neurol. (2012) 46:101–5. 10.1016/j.pediatrneurol.2011.11.007 22264704

[23] Gao P Wang F Huo J Wan D Zhang J Niu J. ALG13 deficiency associated with increased seizure susceptibility and severity. Neuroscience. (2019) 409:204–21. 10.1016/j.neuroscience.2019.03.009 30872163

[24] Bobby GN Erik AE Sergey AS Yin YD Mary-Alice A Carla A. Predominant and novel *de novo* variants in 29 individuals with ALG13 deficiency: clinical description, biomarker status, biochemical analysis, and treatment suggestions. J Inherit Metab Dis. (2020) 43:1333–48. 10.1002/jimd.12290 32681751 PMC7722193

[25] Li D Bhoj E McCormick E Wang F Snyder J Wang T. Early infantile epileptic encephalopathy in an STXBP1 patient with lactic acidemia and normal mitochondrial respiratory chain function. Case Rep Genet. (2016) 2016:1–5. 10.1155/2016/4140780 27069701 PMC4812228

[26] Li T Cheng M Wang J Hong S Li M Liao S. *De novo* mutation of STXBP1 in Chinese children with early onset epileptic encephalopathy. Gene Brain Behav. (2018) 17:e12492. 10.1111/gbb.12492 29896790

